# AHL-based QS signalling promotes uropathogenic *Escherichia coli* settlement through the de-repression of biofilm formation by SdiA

**DOI:** 10.1371/journal.pone.0328837

**Published:** 2025-09-09

**Authors:** Celia Mayer, Fadi Soukarieh, Manuel Simões, Saskia-Camille Flament-Simon, Miguel Cámara, Manuel Romero

**Affiliations:** 1 National Bioﬁlms Innovation Centre, Biodiscovery Institute, School of Life Sciences, University of Nottingham, Nottingham, United Kingdom; 2 LEPABE—Laboratory for Process Engineering, Environment, Biotechnology and Energy, Faculty of Engineering, University of Porto, Porto, Portugal; 3 Laboratorio de Referencia de *E. coli*, FIDIS—Instituto de Investigación Sanitaria de Santiago de Compostela, Santiago de Compostela, Spain; 4 Chemistry, School of Mathematical and Physical Sciences, The University of Sheffield, Sheffield, United Kingdom; 5 School of Allied Health Professions, Pharmacy, Nursing and Midwifery, The University of Sheffield, Sheffield, United Kingdom; 6 ALiCE—Associate Laboratory in Chemical Engineering, Faculty of Engineering, University of Porto, Porto, Portugal; 7 Departamento de Bioquímica y Biología Molecular, Universidade de Santiago de Compostela, Campus Terra, Santiago de Compostela, Spain; 8 Department of Microbiology and Parasitology, Faculty of Biology - Aquatic One Health Research Center (iARCUS), Universidade de Santiago de Compostela, Santiago de Compostela, Spain; Amity University, INDIA

## Abstract

Uropathogenic *Escherichia coli* (UPEC) are among the first pathogens to colonise in catheter and non-catheter-associated urinary tract infections. However, these infections are often polymicrobial, resulting in multi-species infections that persist by forming biofilms. Living within these highly antimicrobial tolerant communities, bacteria can establish intra- and inter-specific interactions, including quorum sensing (QS)-mediated signalling mechanisms, which play a key role in biofilm establishment and maturation. Although *E. coli* does not produce *N*-acylhomoserine lactones (AHLs), it possesses an orphan LuxR-type receptor, SdiA, which can bind these QS signals released by other Gram-negative bacteria, modulating several virulence-associated phenotypes including biofilm formation. Despite biofilms being considered a major public health challenge due to their persistence and resilience, the knowledge of the SdiA role in biofilm regulation and UPEC fitness in mixed biofilms is limited compared to enteropathogenic *E. coli*. We have used a Δ*sdiA* mutant and phenotypic analysis to investigate the SdiA influence on UPEC single and mixed biofilms with *Pseudomonas aeruginosa*. SdiA was found to inhibit UPEC biofilm and addition of AHLs enhanced *E. coli* surface colonisation via SdiA-mediated de-repression of biofilm. We also confirmed the low specificity of SdiA for AHLs, demonstrating the SdiA importance in tightly regulating the UPEC free-living and biofilm-associated lifestyles.

## Introduction

*E. coli* is a Gram-negative commensal bacterium and the most abundant facultative anaerobic microorganism associated with the human and animal intestinal microflora [1], providing many benefits to its hosts as a symbiotic partner [2]. However, several *E. coli* strains or serotypes are also pathogens responsible for serious infections [3]. Indeed, uropathogenic *E. coli* or UPEC are the most common cause of urinary tract infections (UTIs) worldwide, with a total of 404.61 million cases reported in 2019 [4,5]. In addition, UPEC strains are a major cause of nosocomial infections due to their ability to form biofilms on all types of surfaces, including indwelling medical devices, causing serious problems in healthcare facilities [6–8]. Furthermore, treatment of infections caused by these strains is complicated by the fact that some UPEC have developed resistance to several classes of antibiotics used to treat human and animal infections [2]. Indeed, some multidrug-resistant *E. coli* strains have been ranked second on the global priority list of antibiotic-resistant pathogenic bacteria for which new treatment alternatives are urgently needed [9]. According the last 2021 Global Burden of Disease study, *E. coli* is the pathogen with the highest AMR-related mortality rate [5]. Unfortunately, the global burden of UTIs is rising and is influenced by geographical location, age, sex, and economic development, according to the last predictions [5].

Although UPEC and other *Enterobacteriaceae* are among the first microorganisms to colonise the urinary tract, both in UTIs and catheter-associated urinary tract infections (CAUTIs) [10,11], multiple species can colonise the urinary tract over time, leading to mixed-species interactions [12]. Given the polymicrobial nature of these infections, microbial communication through quorum sensing (QS) is thought to play an important role in these inter-species interactions [13]. This chemical communication is based on the production of extracellular signalling molecules called autoinducers. The detection of these autoinducers enables communities of microbial producers, and sometimes non-producer communities, to alter their gene expression in response to changes in population composition and synchronise the production of virulence traits [14]. Although *E. coli* is unable to synthesise *N*-acylhomoserine lactones (AHLs), it possesses an orphan LuxR-type AHL receptor protein, known as SdiA (suppressor of cell division inhibitor), which can respond to these QS molecules released by other Gram-negative bacterial species [15–17]. Interestingly, the SdiA-QS system has been described to play a role in several virulence-associated phenotypes in *E. coli*, including controlling the expression of genes involved in biofilm formation [18].

Biofilms represent a major public health challenge due to their high resistance to conventional therapy, resulting from the action of multifactorial mechanisms which include the limited antimicrobial penetration through the biofilm and up-regulation of resistance pathways by the coloniser *E. coli* [19,20]. Despite the importance of biofilm formation in UPEC, the effect of SdiA on the development of sessile communities in this subset of pathogenic *E. coli* has been much less studied than in enteropathogenic strains. This, in conjunction with the limited reproducibility of conventional biofilm culture methods, particularly when applied to UPEC, results in a significant gap in the field that needs to be addressed. Furthermore, the extant literature provides no definitive conclusion regarding the response of *E. coli* biofilms to exogenous AHLs [21], and research examining the regulatory function of QS in *E. coli* in the context of mixed biofilms is lacking. The objective of this research is to address this knowledge gap by characterising the impact of SdiA on this clinically relevant virulence trait using a UPEC reference strain. The present study employs a rolling biofilm bioreactor (RBB), which has been demonstrated to facilitate robust and reproducible biofilm formation, and produces conditions more representative of those found in the urinary tract. This methodology is especially advantageous when studying mixed-species biofilms, as it facilitates the reproducible formation of complex microbial communities.Here we investigated the role of SdiA from a UPEC strain in biofilm formation in isolation and in co-culture with the opportunistic UTI-associated pathogen *Pseudomonas aeruginosa* which produces AHLs that can be detected by SdiA. This regulator was found to negatively affect biofilm formation in single-species biofilms of UPEC. Addition of exogenous AHLs promoted *E. coli* adherence through the de-repression of biofilm formation via SdiA, thereby enhancing its ability to compete for surface colonisation. Furthermore, our results support the promiscuity of SdiA towards exogenous AHLs, with biological implications for interspecies signalling. This study demonstrates the importance of SdiA in the regulation of free-living and biofilm-associated lifestyles of UPEC strains, addressing both the gap in strain-specific data and the limitations of previous biofilm assays and offering a novel and significant contribution to the understanding of biofilm regulation in UPEC.

## Materials and methods

### Bacterial strains and culture conditions

Bacterial strains and plasmids used in this study are listed in **Table 1**. *E. coli* and *P. aeruginosa* strains were routinely grown at 37ºC in Luria-Bertani (LB) broth and agar supplemented with kanamycin 25 μg/ml, or gentamicin 20 μg/ml, as required. When required, AHLs were synthesised in-house at the University of Nottingham as described before [22], were added to the culture media at a final concentration of 1 or 10 µM from a stock solution prepared in DMSO. The AHLs tested included signals with acyl chains ranging from 4 to 14 carbon atoms and with or without a keto substitution on the third carbon atom of their acyl chain. The molecule NCC40–8841 was obtained from Vitas M laboratory (Hong Kong, China) (S1 Fig) and utilised at a concentration of 100 μM when required. NCC40–8841 was previously identified as a LuxR ligand following a virtual screening process that employed SdiA from *E. coli* as a model LuxR-type receptor and an *in vitro* screening using a *P. aeruginosa* *PrhlI*-*lux* reporter (Soukarieh et al., in preparation).

**Table 1:** Bacterial strains, plasmids and oligonucleotides used in this study.

Strains	Description	Source, reference or usage
***Escherichia coli***		
CFT073	Blood and urine of hospitalised patient with acute pyelonephritis (ST73, O6:K2:H1)	[23]
CFT073 Δ*sdiA*	*sdiA* deletion mutant	[23]
CFT073 pME6032-*gfp*	GFP-labelled CFT073 strain	This study
CFT073 Δ*sdiA* pME6032-*gfp*	GFP-labelled CFT073 *sdiA* mutant strain	This study
CFT073 Δ*sdiA* pME6032-*mCherry*	mCherry-labelled CFT073 *sdiA* mutant strain	This study
CFT073 pME6000-*PftsQ-lux*	CFT073 strain carrying the *PftsQ-luxCDABE* transcriptional reporter in the pME6000 vector	This study
CFT073 Δ*sdiA* pME6000-*PftsQ*-*lux*	CFT073 *sdiA* mutant strain carrying the *PftsQ-luxCDABE* transcriptional reporter in the pME6000 vector	This study
CFT073 pME6000-*PfimA*-*lux*	CFT073 strain carrying the *PfimA-luxCDABE* transcriptional reporter in the pME6000 vector	This study
CFT073 Δ*sdiA* pME6000-*PfimA*-*lux*	CFT073 *sdiA* mutant strain carrying the *PfimA-luxCDABE* transcriptional reporter in the pME6000 vector	This study
DH5α	*E. coli* cloning strain. *F– endA1 glnV44 thi-1 recA1 relA1 gyrA96 deoR nupG purB20* φ*80dlacZ*Δ*M15* Δ*(lacZYA-argF) U169, hsdR17(rK–mK+),* λ*–*	[24]
CM17	UPEC clinical isolate from urine sample. ST131, O25:H4 serotype. B2 phylogenetic group, C2 clade.	HULA collection, [25]
**‍**CM17 pME6000-*PftsQ-lux*	CM17 clinical isolate carrying the *PftsQ-luxCDABE* transcriptional reporter in the pME6000 vector	This study
***Pseudomonas aeruginosa***		
PAO1	*Pseudomonas aeruginosa* PAO1-Nottingham subline	[11]
PAO1 pME6032-*mCherry*	mCherry-labelled PAO1 strain	This study
PAO1 *lasRI::Gm*^*R*^ *rhlRI::Tc*^*R*^	Double mutant of *lasRI*, *rhlRI* QS systems of PAO1 generated using plasmids pSB219.7A and pSB280.1H from [26]	This study
**Plasmid**		
pME6000-*lux*	pME6000 (pBBR1MCS-derived broad host range multicopy vector) containing the *luxCDABE* operon inserted between PstI/XhoI restriction sites, Tc^R^	Construction of *lux* reporter strains
pME6000-*PftsQ-lux*	pME6000 containing a *PftsQ-lux* transcriptional reporter fusion, Tc^R^	This study
pME6000-*PfimA-lux*	pME6000 containing *PfimA-lux* reporter fusion, Tc^R^	This study
pME6032-*gfp*	pME6032Δ*lacI* constituvely expressing GFP, Gm^r^	Construction of GFP fluorescent strains
pME6032-*mCherry*	pME6032Δ*lacI* constituvely expressing mCherry, Gm^r^	Construction of mCherry fluorescent strains
**Oligonucleotide**	**Sequence**	
pftsQFwd	TATGAGCTCGGTCGCGCCGTGGGTAGCGTTA	
pftsQRev	ATACTGCAGATTAGTCCGCCAGTTCCAGAAT	
PfimAFwd	TATGAGCTCCAAGACAATTGGGGCCAAACTG	
PfimARev	ATACTGCAGCCGACAGAACAACGATTGC	

Fwd and Rev: forward and reverse PCR primers respectively. The nucleotide sequences of the restriction sites are underlined.

*HULA: Hospital Universitario Lucus Augusti, Lugo (Spain).

### Transcriptional reporter fusions

Two fragments upstream of the *ftsQ* and *fimA* genes, 594-bp and 478-bp in length, respectively, were amplified by PCR using the oligonucleotides listed in **Table 1**. PCR products were digested with SacI and PstI and ligated into pME6000-*lux*. The resulting plasmids, designated pME6000-*PftsQ*-*lux* and pME6000-*PfimA*-*lux*, were electroporated and propagated into *E. coli* DH5α. Subsequently, plasmids were purified and transformed into UPEC strains. This process led to the generation of strains: *E. coli* CFT073 pME6000-*PftsQ*-*lux*, *E*. *coli* CFT073 Δ*sdiA* pME6000-*PftsQ*-*lux*, *E. coli* CM17 pME6000-*PftsQ*-*lux*, *E. coli* CFT073 pME6000-*PfimA*-*lux*, and *E. coli* CFT073 Δ*sdiA* pME6000-*PfimA*-*lux*, in which the promoter and 5′ regulatory sequences of the *ftsQ* and *fimA* genes (positions −593 to −1 upstream of *ftsQ* and −441 upstream to +18 downstream of *fimA* relative to the start codons) drive the expression of the *lux* operon. Clones with active fusions were selected and analysed for bioluminescence output activity over growth in LB at 37°C using a 96-well plate TECAN Infinite M200 PRO multifunction microplate reader.

### Biofilm assays

Biofilms were cultivated on cover slips (20 mm Ø, Menzel-Glaser, Thermo Scientific) using the rolling biofilm bioreactor (RBB, [27]). Fluorescently tagged strains were inoculated at OD_600 nm_ 0.01 in LB medium and incubated in the RBB system for 24 h and 48 h at 30ºC. Cover slips were examined directly under a laser scanning confocal fluorescent Microscope (Leica TCS SP 5) using excitation wavelengths of 488 nm and 561 nm for eGFP and mCherry respectively. Imaging was carried out using LAS AF (Leica Microsystems, Germany). For colony forming unit (CFU) counts, biofilms were washed once by immersion in phosphate buffered saline (PBS) buffer and disrupted in PBS by sonication for 15’ in a water bath to recover bacterial cells in a Falcon tube and enumerated using the Miles-Misra method [28].

### Transmission electron microscopy analysis

*E. coli* CFT073 wild-type and the Δ*sdiA* mutant were examined by transmission electron microscopy (TEM) to confirm the presence of surface appendages. *E. coli* strains were grown overnight in Müller-Hinton medium at 37ºC without agitation to promote the expression of piliated phenotype. Cultures were then grown in fresh medium under the same conditions for 24 h. Bacterial cells were adjusted to the same optical density, harvested by centrifugation, washed with PBS buffer and fixed with ice-cold 3.5% paraformaldehyde for 1 h. Bacteria were applied to Formvar-coated grids and air-dried. At least two different formvar coated grids were prepared from three different experiments. The cells were then negatively stained with 1% phosphotungstic acid in distilled water for 5 s and examined with a JEM-1011 transmission electron microscope (JEOL JEM-1011) operated at 80 kV and equipped with an Olympus Megaview III (SIS) camera.

### Motility assay

Swimming motility of *E. coli* strains was studied as previously described [29]. Briefly, motility was examined on YLB agar (0.25% agar w/v) with or without AHL supplementation (1 or 10 μM) using 90x15 mm dishes. Swimming plates were inoculated in the center with a microliter drop from agitated overnight cultures at 0.3 optical density (OD_600nm_) and incubated at 37ºC for 16 h. Three plates were prepared for each condition, and experiments were repeated three times.

### Capsule production

The capsule-producing phenotype of *E. coli* CFT073 was evaluated according to methodology previously outlined [30]. Briefly, overnight cultures of wild-type and the Δ*sdiA* mutant of *E. coli* CFT073 were adjusted to an OD_600 nm_ of 1 in 2 mL PBS. The bacterial suspension was overlaid onto a Percoll density gradient comprising 80, 60, 40 and 20% solutions in PBS in order to separate the bacterial fractions after centrifugation (2600 g) at 4°C/20 min (9x acceleration; 1x deceleration). The distance between the bottom of the tube and the migration of the bacterial cell layer was measured.

### Statistical analysis

In order to ascertain whether there were significant differences in the response between bacterial strains and treatments when compared with the variations within the replicates, two-tailed Student’s t-tests were performed. Statistical significance was achieved when *p* < 0.05. The statistical analysis and data plotting were conducted utilising GraphPad Prism 8.0 (GraphPad Software, San Diego, CA, USA).

## Results and discussion

### SdiA modulates biofilm formation in the *E. coli* UPEC strain CFT073

There are conflicting reports in the literature regarding the role of SdiA in biofilm formation in intraintestinal and uropathogenic *E. coli* strains [18]. Several studies describe SdiA as a repressor of biofilm formation in enteric *E. coli* strains [16,31–33] whereas others as a positive regulator of this phenotype in UPEC strains [23,34]. In this study, we used the Rolling Biofilm Bioreactor (RBB) as an optimised culture system for bacterial biofilm formation [27] to evaluate the effect of SdiA on *E. coli* CFT073 biofilm as a reference UPEC strain. In contrast to conventional biofilm models, the RBB system provides a standardised platform for consistently analysing the spatial organisation and architecture of bacterial biofilms [27].

**Fig 1:**
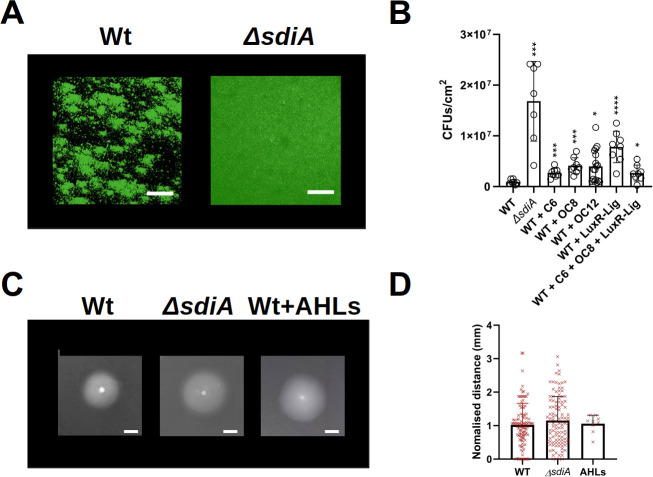
Effect of *sdiA* mutation on biofilm formation and swimming motility in *E. coli* CFT073. **A)** Representative confocal microscopy images comparing biofilm development of GFP-labelled wild-type and GFP-labelled Δ*sdiA* mutant strains of *E. coli* CFT073 after 24 h incubation in the RBB. Volumetric 3D projections of the biofilms were generated using Fiji-ImageJ 1.53 [36]. Scale bar: 50 μm. **B)** Number of viable cells (CFU/cm^2^) in *E. coli* CFT073 wild-type and the Δ*sdiA* mutant biofilms grown for 24 h culture in LB at 30ºC. C6, OC8 and OC12-HSL were added separately to a final concentration of 1 μM, and NCC40-8841 molecule was added to a final concentration of 100 μM separately (WT + LuxR-Lig) or in combination with C6 and OC8-HSL at 1 μM each (WT + C6 + OC8 + LuxR-Lig). **C)** and **D)** representative images and distances of swimming motility plates (YLB supplemented with 0.25% agar) of *E. coli* CFT073 wild-type, Δ*sdiA* mutant, and wild-type with AHLs (C6 and OC8-HSL) after 16 h incubation. Scale bar: 10 mm. Data shown are mean ± SD. Statistics are two-tailed Student’s t-test (*p < 0.05; **p < 0.01; ***p < 0.001; ****p < 0.0001) (GraphPad Prism 8.0).

Although CFT073 is a poor biofilm-former in commonly used *in vitro* biofilm models, this is not the case in the RBB system, where biofilms of this strain are reproducibly formed enabling robust quantification of biofilm parameters (**Fig 1A**). Consistent with reports describing SdiA as a repressor of biofilm in *E. coli*, our results showed that a Δ*sdiA* mutant made in CFT073 produced a significantly higher amount of biofilm compared to the wild-type strain after 24 h of culture in the RBB system (**Fig 1A**).

As demonstrated in previous studies, SdiA has been found to impede the function of division inhibitors and induce minicell formation in *E. coli* [35]. In order to ascertain whether SdiA deficiency in CFT073 could also impact cell division and growth, and thereby provide an explanation for the observed differences in biofilm formation, the cell size and growth in both the wild-type and the Δ*sdiA* mutant strains of CFT073 were studied. A comparison of cell size revealed that the Δ*sdiA* mutant exhibited a slight reduction in size compared to the wild-type (S2B Fig). However, no disparities in growth rates were detected (S2A Fig), thereby excluding this mechanism as a contributing factor to the observed variations in biofilm formation.

**Fig 2:**
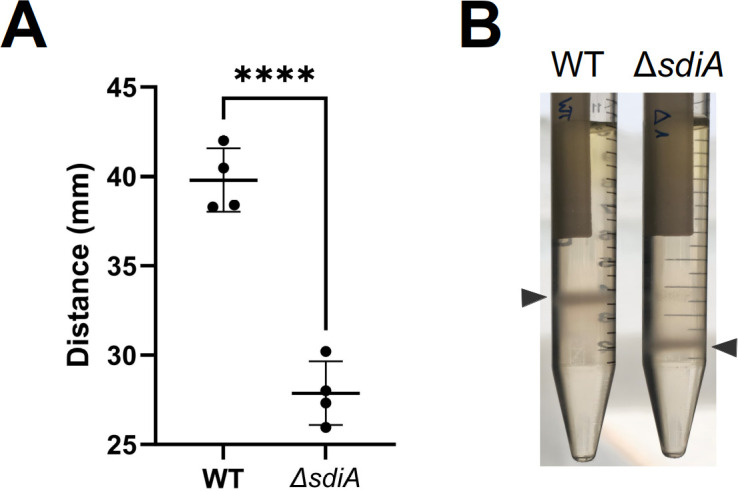
Percoll density gradient assay with *E. coli* CFT073. **A)** Cultures of wild-type (left) and Δ*sdiA* (right) strains of CFT073 were placed in a Percoll gradient. Macroscopic capsule modification in the Δ*sdiA* mutant was confirmed by an increase in bacterial migration through the Percoll gradient after centrifugation in comparison to the wild-type strain. Cells are visualised as an opaque band indicated by arrowheads. **B)** Graph representing the height measurements of the bacterial cell layers (N = 4).

Recent studies conducted in our laboratory have demonstrated that capsule-related phenotypes are influenced by a *sdiA* mutation in *Klebsiella pneumoniae* [37] and capsule polysaccharides have been suggested to exert a negative impact on biofilm, as evidenced by the observation that high-capsule-producing bacteria exhibit deficient biofilm formation [38]. In order to explore the mechanism underlying the differences between wild-type and Δ*sdiA* mutant biofilm formation, the capsule production of both strains was assessed using Percoll density gradient centrifugation. This method facilitates the macroscopic differentiation of high and low capsule-producing bacteria based on their flotation characteristics [30]. The results demonstrated a discernible distinction between the wild-type strain and the Δ*sdiA* mutant with respect to capsule production, exhibiting a clear reduction in capsule in the latter (**Fig 2**). As described in the literature, this finding suggests a correlation between low capsule production and high biofilm formation by SdiA deficiency in CFT073. Further research is warranted to explore this association more thoroughly.

To determine whether the presence of AHLs could affect biofilm formation by CFT073, the synthetic quorum sensing (QS) signals *N*-hexanoyl-L-homoserine lactone (C6-HSL) and *N*-3-oxo-octanoyl-L-homoserine lactone (OC8-HSL) were added to the biofilm cultures. These QS signals were chosen because previous studies have described that the SdiA regulon responds specifically to exogenous AHLs with 6- to 8-carbon acyl chains and keto modifications at the third carbon [39,40]. In addition, the long-chain AHL *N*-3-oxododecanoyl-L-homoserine lactone (OC12-HSL) was also tested. Moreover, to further investigate the role of SdiA in biofilm formation by CFT073, the molecule NCC40–8841, a LuxR-QS inhibitor, was also added to the biofilm assays. This molecule was discovered through *in-silico* screening on a LuxR-type QS receptor binding domain and was found to bind to this receptor at low micromolar concentration, as determined by structure-based and biological evaluation (Soukarieh et al., in preparation).

As with the Δ*sdiA* mutant, and in contrast to previous studies showing a reduction in biofilm formation by enteric *E. coli* strains following the addition of exogenous AHLs [31,40], quantification of viable biofilm cells cultured in the RBB system revealed a significant increase in biofilm production by CFT073 in the presence of all AHLs tested (**Fig 1B**). Interestingly, our results also showed an increase in CFT073 biofilm production upon addition of the LuxR ligand NCC40–8841 (**Fig 1B**), suggesting that this compound, similar to AHLs, could reverse the role of SdiA in suppressing *E. coli* CFT073 biofilm formation. Likewise, a significant but comparatively minor increase in biofilm formation was observed upon addition of both the LuxR ligand and AHLs together (**Fig 1B**). This phenomenon could be ascribed to the exogenous AHLs competing with the LuxR ligand for SdiA binding, thereby augmenting biofilm production to a lesser extent in comparison with the ligand-only treatment. Nevertheless, further experimentation is required to verify this hypothesis.

SdiA can bind a wide range of signals that affect its stability and ability to regulate transcription [16,17]. Therefore, it is possible that binding of SdiA to AHLs or to other LuxR ligands could block SdiA activity and thus prevent its repressor role in biofilm formation. Alternatively, AHLs could compete with the binding of SdiA to unknown non-AHL cognate signals that induce biofilm suppression in *E. coli*. However, it should be noted that the effect of compound NCC40–8841 on CFT073 biofilm formation in our study was opposite to that of the potential SdiA ligand fructose-furoic acid ester used in a previous study, which showed a decrease in average thickness and biomass biofilm after addition, and this was attributed to competition for binding to the AHL *N*-octanoyl-L-homoserine lactone (C8-HSL) by SdiA [34]. In *E. coli*, the switch from a planktonic to a biofilm lifestyle is regulated by inversely controlled regulatory cascades whose final outputs of flagellar production or synthesis of biofilm matrix components/fimbriae are mutually exclusive [41]. Given that SdiA negatively modulates biofilm formation in CFT073 (**Fig 1A**), we measured swimming motility in the Δ*sdiA* mutant and wild-type strains to assess whether SdiA could also affect this phenotype and thus influence the balance between motility and sessility in this UPEC. Consistent with previous studies of CFT073 motility [23], no significant differences in swimming were observed between wild-type and Δ*sdiA* strains (**Fig 1C and 1D**), suggesting that SdiA has no apparent effect on this motility, in contrast to biofilm formation. Furthermore, the addition of AHLs had no effect on the motility of the wild-type strain (**Fig 1C and 1D**), further indicating that exogenous AHL signalling does not significantly alter the motility of CFT073 under the conditions tested.

### SdiA and exogenous QS signals promote *E. coli* biofilm growth in mixed bacterial populations

**Fig 3:**
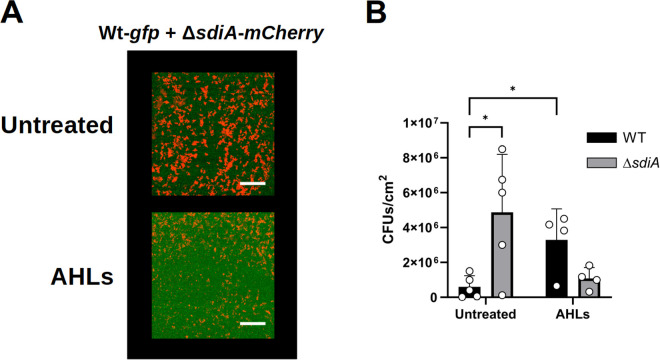
Effect of *sdiA* mutation on biofilm formation in co-cultures with wild-type strain of *E. coli* CFT073. **A)** Representative confocal microscopy images of biofilms produced by *E. coli* CFT073 wild-type strain (green, GFP-tagged) co-cultured with the Δ*sdiA* mutant (red, mCherry-tagged). 3D projections of biofilms were generated using the open-source software Fiji-ImageJ 1.53 [36]. Scale bar: 50 μm. The “AHLs” label indicate that the signals C6 and OC8-HSL were added at 10 µM each. **B)** Colony forming units (CFU/cm^2^) from biofilms of *E. coli* CFT073 wild-type and Δ*sdiA* mutant strains co-cultured in the RBB system for 24 h. Data shown are mean ± SD (n = 6). Statistics are two-tailed Student’s t-test (*p < 0.05; **p < 0.01; ***p < 0.001; ****p < 0.0001) (GraphPad Prism 8.0).

Bacteria in the clinical and natural environment tend to live in polymicrobial communities, and their behaviour in mixed-species biofilms can significantly differ from those in single-species biofilms [42]. In the urinary tract, UPECs interact with other bacterial strains and species with which they coexist or compete to colonise the host. Despite this, few reports have explored the microbial interaction between clinical uropathogenic *E. coli* strains and other potential pathogenic bacterial species from the urinary tract. To determine the effect of SdiA in the context of competition for surface colonisation, biofilm formation by mixed bacterial populations was studied using fluorescently labelled strains. First, biofilms of wild-type and Δ*sdiA* mutant CFT073 strains were co-cultured with or without supplementation with AHL signals (C6 and OC8-HSL) and adherent cells from both populations were quantified. Consistent with our previous results, the mutant (mCherry-labelled) population outcompeted the wild-type (GFP-labelled) population, with the mutant producing significantly more biofilm and almost completely displacing the wild-type (89% of the total) in mixed biofilms. However, in the mixed biofilm, the Δ*sdiA* mutant was found to have a reduced capacity to colonise the entire surface, in contrast to its behaviour in monoculture (**Fig 3**). Remarkably, the addition of AHLs completely reversed these results, with the wild-type strain representing a much higher percentage of the total biofilm population in the co-cultures, suggesting that the ability of the CFT073 strain to sense exogenous AHL signals may enhance its ability to compete for surface colonisation (**Fig 3**). Furthermore, these results again seem to indicate that the presence of AHL-type QS signals promotes the adherence of *E. coli* through de-repression of biofilm formation by SdiA.

SdiA is conserved across various genera within the *Enterobacteriaceae* family, including *Salmonella*, *Enterobacter*, *Citrobacter*, and *Klebsiella* [43]. However, SdiA has also been observed in bacteria of a different taxonomic classification, including those isolated from humans or terrestrial environments [17,21]. In these cases, *sdiA* has been found to be conserved in all lineages, suggesting a crucial role common to these organisms, despite their diverse specific niches [21]. Interestingly, a similar behaviour to that exhibited by *E. coli* has been observed in the context of biofilm formation in *K. pneumoniae*, where AHL supplementation enhances biofilm formation when SdiA is present and, in a manner analogous to UPEC, SdiA deficiency was observed to increase biofilm formation in this species [37].

**Fig 4:**
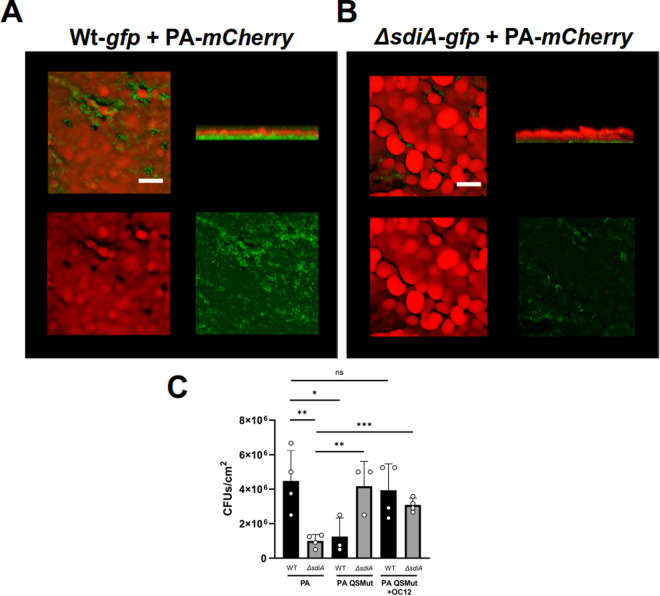
Biofilm competition assays between *P. aeruginosa* (PA) and *E. coli* CFT073. **A)** Representative confocal microscopy images of 24 h biofilms produced by *E. coli* CFT073 wild-type and **B)** Δ*sdiA* mutant strains (green, GFP-tagged) co-cultured with *P. aeruginosa* PAO1 (red, mCherry-tagged). (1: composite image; 2: lateral volumetric 3D projection of the biofilms; 3 and 4: red and green channels, respectively). Mushroom-like structures produced by *P. aeruginosa* are located at the air-liquid exposure layers, while discrete *E. coli* cell clusters are shown at the bottom of the mixed biofilm. 3D projections of the biofilms were generated using the open-source software Fiji-ImageJ 1.53 [36]. Scale bar: 50 μm. **C)** Colony forming units (CFU/cm^2^) of *E. coli* CFT073 wild-type and Δ*sdiA* mutant strains from 24 h biofilms co-cultured with *P. aeruginosa* PAO1 wild-type (PA) or *lasRI::Gm*^*R*^ *rhlRI::Tc*^*R*^ mutant (QSMut) with or without addition of 1 μM OC12-HSL. Data shown are mean ± SD. Statistics are two-tailed Student’s t-test (*p < 0.05; **p < 0.01; ***p < 0.001; ****p < 0.0001) (GraphPad Prism 8.0).

To assess whether the ability of SdiA to regulate biofilm formation in response to the presence of exogenous QS signalling could provide an advantage to *E. coli* in the context of dominance in mixed populations with other bacterial species present in the urinary tract, we also examined biofilm formation between CFT073 and *Pseudomonas aeruginosa*, a human opportunistic pathogen commonly associated with urinary tract infections [44–46]. In addition, and to specifically determine the effect of *P. aeruginosa* QS signalling on SdiA-mediated biofilm formation, the *lasRI::Gm*^*R*^ *rhlRI::Tc*^*R*^ mutant strain of *P. aeruginosa*, disabled in AHL-based QS communication due to a lack of AHL synthases and receptors, was also included in the assays. Despite the high biofilm-forming capacity and rapid surface colonisation exhibited by wild-type *P. aeruginosa* in the RBB model (including the formation of the characteristic mushroom-shaped structures in biofilms of this bacterium under certain conditions – [47]), both species were able to coexist in mature (48–72 h) biofilms, with *E. coli* wild-type forming a monolayer of cell aggregates at the bottom of the dual-species community (**Fig 4A**). In contrast, when the Δ*sdiA* mutant was co-cultured with *P. aeruginosa* wild-type, there was a significant decrease in the amount of biofilm produced by this Δ*sdiA* mutant (**Fig 4B**) compared to its *E. coli* parental strain (**Fig 4C**). In both cases, *P. aeruginosa* continues to form the characteristic three-dimensional structures, which appear slightly larger when co-cultured with the Δ*sdiA* mutant (**Fig 4A and 4B**).

Quantified CFUs from the dual-species biofilm assay involving CFT073 and *P. aeruginosa* are shown in **Fig 4C**. Interestingly, and in agreement with our previous results in mixed biofilms where the Δ*sdiA* mutant displaced the *E. coli* wild-type population (**Fig 3**), when *E. coli* (wild-type or Δ*sdiA* mutant) was co-cultured with the QS-deficient *P. aeruginosa* strain, which is unable to produce AHL signals, the Δ*sdiA* mutant formed more biofilm compared to its parental strain (**Fig 4C**).

The divergent behaviour exhibited by the Δ*sdiA E. coli* strain in the presence of *P. aeruginosa* wild-type or QS-deficient strains can be attributed, at least in part, to a diminution in the competitiveness of the latter within a heterogeneous bacterial population. This reduction can be attributed to the absence of factors produced under the regulation of QS, which have been demonstrated to subjugate competing bacteria. Thus, there appears to be an additional effect triggered by a QS effector/factor in *P. aeruginosa* that could also have an impact on *E. coli* biofilm formation. Given that OC12-HSL, an AHL produced by *P. aeruginosa,* can cause an increase in biofilm formation by CFT073 in monospecies cultures (**Fig 1B**), mixed cultures of CFT073 strains and the QS mutant of *P. aeruginosa* were also supplemented with OC12-HSL to assess whether this signal mediates the promotion of wild-type CFT073 biofilm formation by *P. aeruginosa*. Results showed that while no significant effect on biofilm formation by the Δ*sdiA* mutant was observed in cultures supplemented with OC12-HSL in the presence of the QS mutant of *P. aeruginosa*, the addition of OC12-HSL to mixed cultures of wild-type CFT073 cells and the QS mutant of *P. aeruginosa* significantly increased adherence of the former (**Fig 4C**), suggesting that the effect of *P. aeruginosa* on CFT073 biofilm could be partly mediated by SdiA sensing this AHL. It should be noted that *P. aeruginosa* also produces the AHL signal C4-HSL. It is therefore advisable that further experiments be conducted using C4-HSL or a combination of C4-HSL and OC12-HSL, since this could enhance the physiological relevance of the findings.

**Fig 5:**
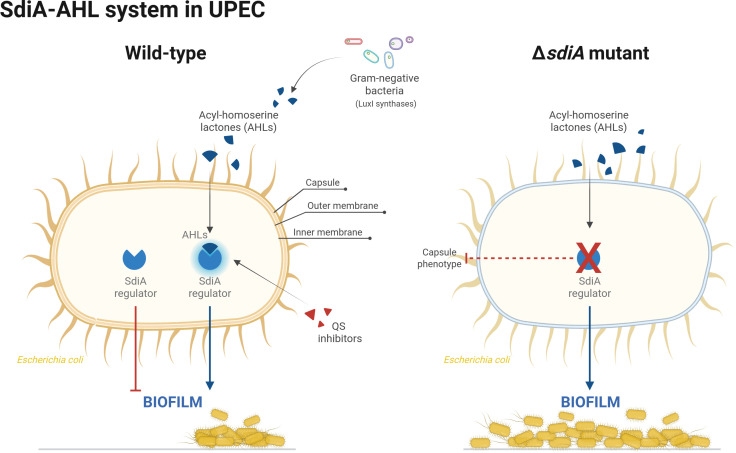
Schematic representation of the LuxR-type QS system in *E. coli.* Despite the absence of a synthase capable of producing AHL signals (blue curved triangles) in *E. coli*, this species has been shown to detect these molecules produced by other Gram-negative bacterial species, including *P. aeruginosa*. This detection is facilitated by the orphan SdiA regulator, which modulates several virulence-associated phenotypes in *E. coli*, including biofilm formation. The results of this study suggest that SdiA exerts a repressive effect on biofilm production and that the presence of AHLs or SdiA deficiency enhances UPEC surface colonisation through de-repression of biofilm formation, possibly through inhibition of capsule production. The blue arrows are used to denote positive regulation, while the red lines are used to denote phenotype inhibition. The dotted red line indicates a potential role in the regulation of the phenotype. Created with BioRender.

Taken together, these results suggest that UPEC can sense QS signalling from wild-type *P. aeruginosa*, stop biofilm suppression by SdiA, and in turn form more biofilm and outcompete the Δ*sdiA* mutant (**Fig 5**). The ability of SdiA to respond to AHLs produced by other bacterial species to modulate the gene expression has been previously observed by several authors [16,48,49]. In addition, orphan LuxR receptors can detect signals produced in eukaryotic hosts, by themselves or by other microbial species [50].

Indeed, *E. coli* can establish interspecific bacterial communication as an adaptive advantage to sense the environment and respond to QS signals produced by cooperating or competing bacteria to adapt to complex environments such as mammalian hosts [13,17,51,52]. In addition, it should be noted that *E. coli* also has an interspecies cell-cell communication system based on the Autoinducer-2 (AI-2) signal. This system has a significant impact on biofilm production [53] and a link has been identified between AI-2 QS and SdiA in *E. coli*, facilitated by the EAL domain protein encoded by *ydiV* and cAMP. However, a direct effect on *sdiA* gene expression by AI-2 has not been found to date [54].

Biofilms are ideal environments for promoting cross-species communication, facilitating the sensing and exchange of small chemical molecules and nutrients. Consistent with our results, coexistence between *E. coli* and *P. aeruginosa* in a dual-species biofilm has also been observed, with the former colonising the interior of *P. aeruginosa* clusters when co-inoculated under different experimental conditions [55–57]. Furthermore, a reduction in mixed-species biofilm formed by *E. coli* and *P. aeruginosa* on urinary catheters coated with QS-inhibiting enzymes has been demonstrated [58], supporting the role of exogenous QS signalling in promoting biofilm formation by *E. coli* in the context of mixed bacterial populations. The utilisation of transcriptomic analysis has the potential to serve as a valuable method for elucidating the mechanism of SdiA regulation in UPEC biofilm formation in response to AHLs. Consequently, acquiring knowledge on the most suitable signals for this receptor would be advantageous for conducting these complementary analyses.

### SdiA from *E. coli* CFT073 can detect multiple AHL signals

The previously observed versatility of LuxR receptors in binding various AHLs would allow the development of cross-species communication networks in emerging biofilms [13,17]. Our results so far suggest that the SdiA-mediated ability of *E. coli* to sense exogenous AHLs and produce more biofilm may provide a competitive advantage for successful surface colonisation in mixed bacterial populations. Therefore, to test the ability of *E. coli* CFT073 to sense different AHLs, we decided to evaluate the specificity of SdiA for a number of these signals using bioluminescence-based reporters whose expression depends on this regulator. The selected genes included the promoter region of the *ftsQAZ* operon, which is involved in cell division and was previously described to be regulated by SdiA in *E. coli* [59], and the *fimA* promoter, a pilin structural gene of type I fimbriae and a key factor in UPEC adherence to host epithelial cells in the urinary tract [60], which expression was previously described to be affected by exogenous AHL addition [33].

**Fig 6:**
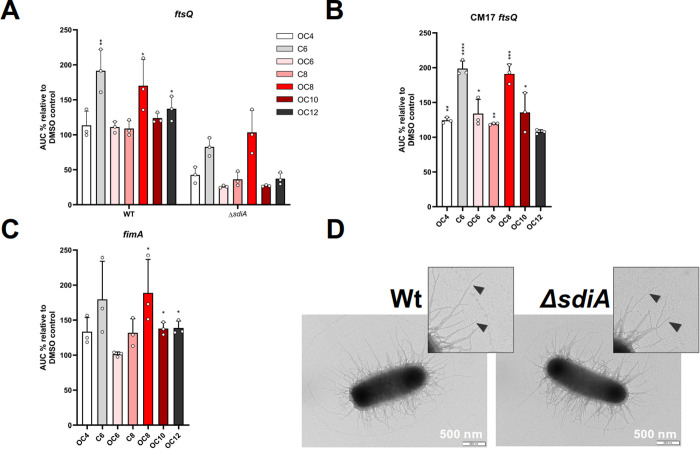
Effect of AHL addition and/or *sdiA* mutation on *ftsQ* and *fimA* transcription and on fimbriae production in UPEC. **(A)** Wild-type and Δ*sdiA* mutant strains of CFT073 carrying the *PftsQ*-*lux* transcriptional fusion, **(B)** UPEC clinical isolate CM17 carrying the *PftsQ*-*lux* transcriptional fusion, and **(C)** wild-type CFT073 carrying the *PfimA-lux* reporter. 10 μM of each AHL or DMSO solvent was added to the culture as a negative control. Data shown correspond to AHLs with positive impact on the transcriptional fusions in the strains. Values given are averages from three different cultures ± standard deviation and correspond to the area under the curve (AUC) derived from plotting relative light units normalised to culture density over time (24 h), and as percentage of the corresponding activity obtained in control cultures (set at 100%). Statistics are two-tailed Student’s t-test (*p < 0.05; **p < 0.01; ***p < 0.001; ****p < 0.0001) (GraphPad Prism 8.0). **(D)** Representative TEM micrographs of *E. coli* CFT073 wild-type and Δ*sdiA* mutant. Both strains expressed large numbers of fimbriae with similar morphology (arrowheads). Scale bar: 500 nm.

As shown in **Figs 6A**, and S3A, the addition of the AHLs C6, OC8, and OC12-HSL resulted in a significant induction of luminescence in the *E. coli* CFT073 bioreporter containing the *PftsQ*-*luxCDABE* transcriptional fusion. However, none of the AHLs produced a significant luminescence induction in the Δ*sdiA* mutant (**Fig 6A**), confirming that SdiA controls the expression of the *ftsQAZ* operon in CFT073 and indicating the low AHL specificity of SdiA in this bacterium. Interestingly, some AHL treatments resulted in reduced luminescence production in the Δ*sdiA* mutant reporter, suggesting an alternative regulatory mechanism independent of SdiA. It is noteworthy that OC12-HSL was one of the activating AHLs in the wild-type strain, providing further support for a role of SdiA in sensing this *P. aeruginosa* signal in CFT073 (**Fig 4**).

The *PfimA-luxCDABE* reporter transformed in CFT073 also showed a similar response to the same AHL signals (**Fig 6C**). Although the increase produced by C6-HSL was not significant, OC8, OC10 and OC12-HSL produced a significant higher luminescence in the wild-type. These results confirm the versatility of the CFT073 SdiA regulator in detecting exogenous QS signals. Although these results support an effect of AHL supplementation on *fimA* expression, our data shows an increase in *fimA* reporter expression, in contrast to previous reports showing a decrease in *fimA* transcription upon addition of exogenous AHL [33]. In light of this, and to assess potential differences between wild-type and Δ*sdiA* mutant strains, the formation of type I fimbriae in *E. coli* CFT073 was examined by transmission electron microscopy (TEM). Despite this, no significant differences in the production of type I fimbriae were found between the two strains (**Fig 6D**). However, it should be noted that the genome of CFT073 is particularly rich in genes encoding putative fimbrial adhesins, with 12 distinct fimbrial gene clusters as revealed by genome sequencing [61], making it difficult to identify changes in surface appendage production in this strain.

In order to evaluate whether the response to AHLs by other UPEC strains differed from that of the CFT073, an attempt was made to transform clinical isolates with the *PftsQ-lux* transcriptional reporter that respond to these signals. The clinical isolates selected belonged to the sequence type 131 (ST131), a fluoroquinolone-resistant lineage that produces CTX-M-14 β-lactamase and is globally recognize as one of the major extraintestinal pathogenic *E. coli* lineages [25]. The successful introduction of the *PftsQ-luxCDABE* transcriptional fusion was achieved in the CM17 strain, responsible of a human UTI. Similarly to CFT073, the strain CM17 exhibited a response to AHLs with an increased induction of luminescence observed for the AHLs C6 and OC8-HSL (**Fig 6B**), suggesting a parallel behaviour of the SdiA regulator in these UPEC strains.

## Conclusions

The results obtained suggest a key role for SdiA in modulating biofilm formation by UPEC. SdiA was found to have a detrimental effect on the production of biofilm in *E. coli* CFT073, and the addition of exogenous QS signals promoted *E. coli* CFT073 surface colonisation via SdiA-mediated de-repression of biofilm formation. As demonstrated in previous studies in *E. coli*, this investigation reveals that the SdiA regulator exhibits low specificity for exogenous AHLs in UPEC. The hypothesis that a functional SdiA regulator confers a competitive advantage to UPEC in the context of mixed bacterial populations in the urinary tract is supported by the results of intra- and inter-species competition experiments *in vitro* using wild-type and *sdiA*-deficient UPEC strains. However, further experimentation is required to confirm the underlying mechanism of SdiA competitiveness promotion in UPEC. In summary, the findings of this study demonstrate the capacity of SdiA to influence the planktonic and biofilm-associated lifestyles of UPEC.

## Supporting information

S1 Fig Chemical structure of NCC40–8841 compound. (TIF)

S2 Fig Growth curves and cell size distribution of *E. coli* CFT073 wild-type and Δ*sdiA* strains. **A)** Growth of CFT073 strains in shaken cultures (200 rpm) of LB incubated at 37ºC. Data shown are mean ± SD. **B)** Distribution of cell size (μm) fractions in biofilms of wild-type and Δ*sdiA* mutant. Cell sizes were measured using the ImageJ software (version 1.54) from microscopy images. A total of 500 cells were analysed for each strain. Statistics are two-tailed Student’s t-test (*p < 0.05; **p < 0.01; ***p < 0.001; ****p < 0.0001) (GraphPad Prism 8.0).
(TIF)

S3 Fig Effect of AHL addition on *ftsQ* and *fimA* transcription in *E. coli* CFT073. Wild-type strain of CFT073 carrying **(A)** *PftsQ-lux* or **(B)** *PfimA-lux* transcriptional fusion. 10 μM of each AHL or DMSO solvent was added to the culture as negative control. Data shown correspond to AHLs with positive impact on the transcriptional fusions in the CFT073 wild type strain. Values in plots represent relative light units (RLU) normalised to culture density (OD_600nm_).
(TIF)

S1 Data Data file from manuscript. (XLSX)
