# Supplementary figures and images for: AHL-based QS signalling promotes uropathogenic Escherichia coli settlement through the de-repression of biofilm formation by SdiA
Source: PLoS One. 2025 Sep 9;20(9):e0328837. doi: 10.1371/journal.pone.0328837 (PMC12419603; doi:10.1371/journal.pone.0328837)

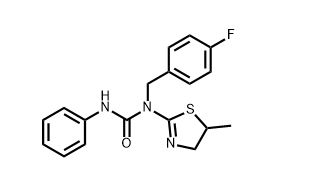

Supplement: S1 Fig — (TIF) [file pone.0328837.s001.tif]

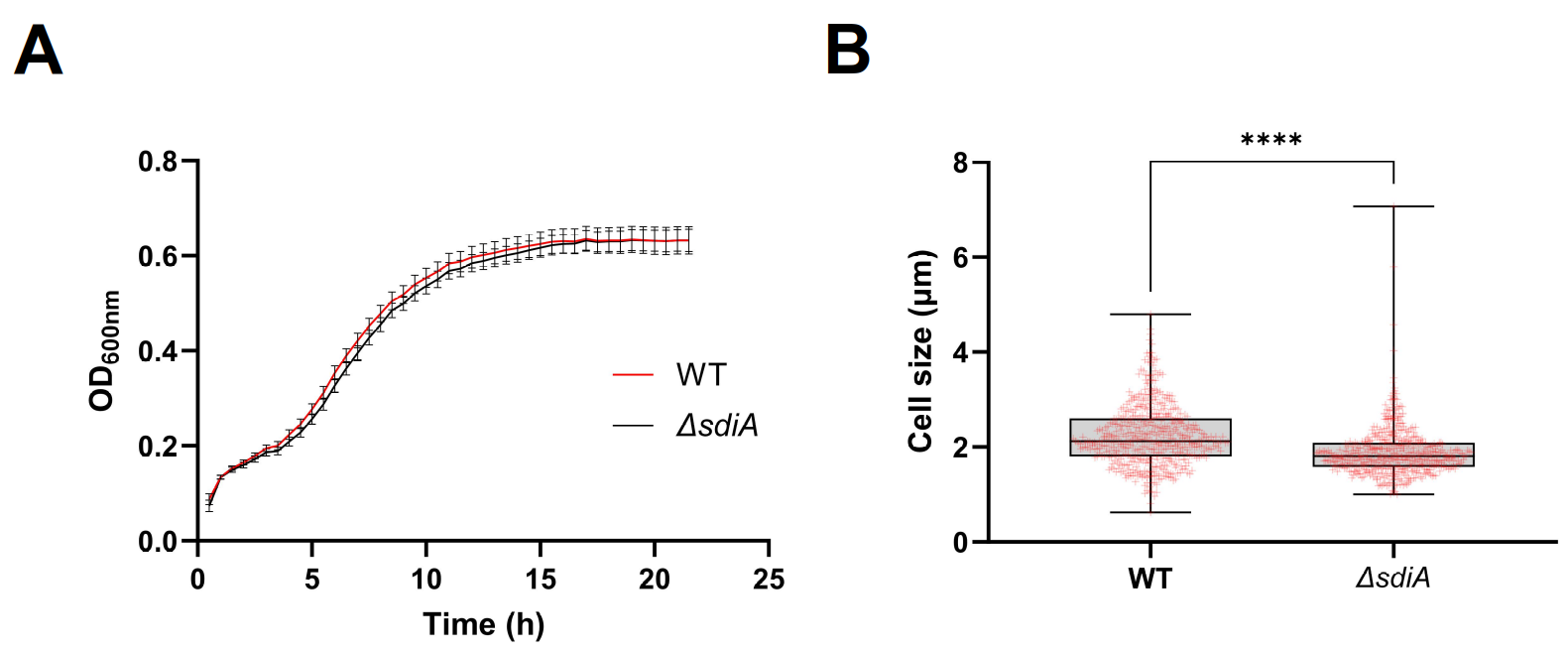

Supplement: S2 Fig — A) Growth of CFT073 strains in shaken cultures (200 rpm) of LB incubated at 37ºC. Data shown are mean ± SD. B) Distribution of cell size (μm) fractions in biofilms of wild-type and ΔsdiA mutant. Cell sizes were measured using the ImageJ software (version 1.54) from microscopy images. A total of 500 cells were analysed for each strain. Statistics are two-tailed Student’s t-test (*p < 0.05; **p < 0.01; ***p < 0.001; ****p < 0.0001) (GraphPad Prism 8.0). (TIF) [file pone.0328837.s002.tif]

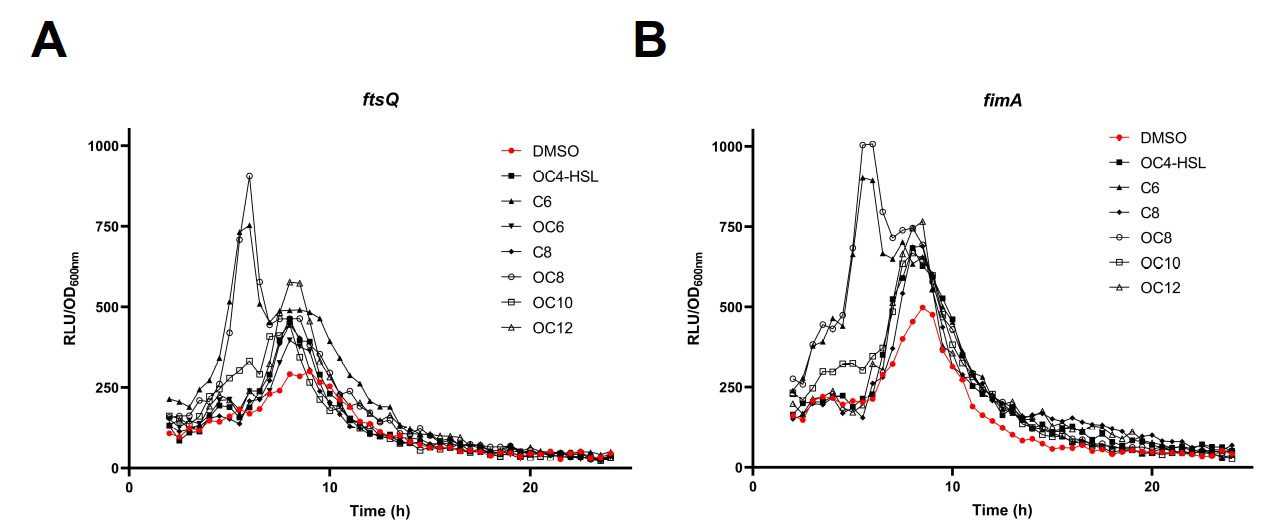

Supplement: S3 Fig — Wild-type strain of CFT073 carrying (A) PftsQ-lux or (B) PfimA-lux transcriptional fusion. 10 μM of each AHL or DMSO solvent was added to the culture as negative control. Data shown correspond to AHLs with positive impact on the transcriptional fusions in the CFT073 wild type strain. Values in plots represent relative light units (RLU) normalised to culture density (OD600nm). (TIF) [file pone.0328837.s003.tif]
